# Nutritional Impact and Its Potential Consequences on COVID-19 Severity

**DOI:** 10.3389/fnut.2021.698617

**Published:** 2021-07-05

**Authors:** Esmaeil Mortaz, Gillina Bezemer, Shamila D. Alipoor, Mohammad Varahram, Sharon Mumby, Gert Folkerts, Johan Garssen, Ian M. Adcock

**Affiliations:** ^1^Department of Immunology, Faculty of Medicine, Shahid Beheshti University of Medical Sciences, Tehran, Iran; ^2^Clinical Tuberculosis and Epidemiology Research Centre, National Research Institute of Tuberculosis and Lung Diseases, Shahid Beheshti University of Medical Sciences, Tehran, Iran; ^3^Division of Pharmacology, Faculty of Science, Utrecht Institute for Pharmaceutical Sciences, Utrecht University, Utrecht, Netherlands; ^4^Impact Station, Hilversum, Netherlands; ^5^Molecular Medicine Department, Institute of Medical Biotechnology, National Institute of Genetic Engineering and Biotechnology, Tehran, Iran; ^6^Mycobacteriology Research Centre, National Research Institute of Tuberculosis and Lung Diseases (NRITLD), Shahid Beheshti University of Medical Sciences, Tehran, Iran; ^7^Airways Disease Section, Faculty of Medicine, National Heart and Lung Institute, Imperial College London, London, United Kingdom; ^8^Center of Excellence Immunology, Nutricia Research, Utrecht, Netherlands

**Keywords:** COVID-19, SARS-CoV-2, probiotics, nutrition, proteins

## Abstract

**Background:** During late 2019 a viral disease due to a novel coronavirus was reported in Wuhan, China, which rapidly developed into an exploding pandemic and poses a severe threat to human health all over the world. Until now (May 2021), there are insufficient treatment options for the management of this global disease and shortage of vaccines. Important aspects that help to defeat coronavirus infection seems to be having a healthy, strong, and resilient immune system. Nutrition and metabolic disorders, such as obesity and diabetes play a crucial role on the community health situation in general and especially during this new pandemic. There seems to be an enormous impact of lifestyle, metabolic disorders, and immune status on coronavirus disease 2019 (COVID-19) severity and recovery. For this reason, it is important to consider the impact of lifestyle and the consumption of well-defined healthy diets during the pandemic.

**Aims:** In this review, we summarise recent findings on the effect of nutrition on COVID-19 susceptibility and disease severity and treatment. Understanding how specific dietary features might help to improve the public health strategies to reduce the rate and severity of COVID-19.

## Introduction

The recent outbreak of coronavirus disease 2019 (COVID-19), caused by a new zoonotic severe acute respiratory syndrome coronavirus 2 (SARS-CoV-2) (1), is a great threat to public health all over the world (2). As of May 20th 2021, variants of the coronavirus SARS-CoV-2 have infected more than 165 million people globally and resulted in 3.42 million deaths (3). Beyond prevalence and mortality, the restrictions and lockdown measures that are needed to control the COVID-19 pandemic evolved in a global economic and social crisis, severely affecting the people's well-being, mental health and social support (4). The direct consequences of COVID-19 on an individual represents a spectrum of clinical severity with some patients being asymptomatic or having only mild upper respiratory tract symptoms whilst some subjects have severe pneumonia characterised by fever, cough, dyspnoea, bilateral pulmonary infiltrates and acute respiratory injury requiring ventilation (5–8). Approximately 20% of patients develop severe respiratory illness with an overall mortality of 2.3% (3). The impact of SARS-CoV-2 infection is not limited to the respiratory system, but it affects the kidney, gut, eyes, heart, and brain among other organs. Together, the effect on these target organs may have profound and prolonged consequences on COVID-19 severity, and on recovery (5–8). The body's mental and physical status and fitness are important factors in keeping one's immune system balanced and resilient and thereby able to mount a proper response against SARS-CoV-2 (9, 10). Obesity and type 2 diabetes are therefore examples of key risk factors for COVID-19 (11). Obesity is associated with dysfunctional adipose tissue, metabolic dysfunction, multi organ damage, endocrine disruption, impaired immune function, and low grade (sub) chronic inflammation (12). Moreover, obesity along with low physical activity and fitness, is the leading cause of type 2 diabetes or metabolic syndrome (T2DM), which is causally linked with elevated angiotensin-converting enzyme 2 (ACE2) expression (13).

The high prevalence of these risk factors, is for a significant part, associated with the pattern of nutrition such as increased consumption of high amounts of saturated fat (high fat diet, HFD), refined carbohydrates and low levels of fibre and antioxidants. Balanced nutrition has a potentially important role in the maintenance of immune homeostasis and resilience and for this reason resistance against disease including infections with viral and bacterial pathogens. Malnutrition has prolonged effects on physical and mental health by influencing gene expression, cell activation, and interfering with signalling molecules that shape and modulate the immune system (14). Thus, poor nutrition and an unhealthy diet might significantly weaken the immune system and increases susceptibility to infectious disease including SARS-CoV-2.

Disparities in nutrition or obesity are impacted by cultural background and closely correlated with severe COVID-19-related outcomes (15). The hospitalisation rates for COVID-19 positive subjects among Native and Latin Americans are higher than that of White Americans which could be attributed to malnutrition (15, 16). Another example of the impact of cultural background and socio-economic status on severity of COVID-19 is evidenced in Islamic countries with poor healthcare systems, lack of facilities particularly during the religious tradition of Ramadan fasting (17). During Ramadan, Muslims may have trouble in maintaining exercise, which negatively affects immune health. On the other hand investigations of health related effects of Ramadan Fasting also show beneficial effects of reduced meal frequency and caloric restrictions on insulin sensitivity, a reduction in oxidative stress and inflammation (17).

Indeed, nutrition and obesity play a crucial role in the fate of viral infectivity in general and the community health situation during this present pandemic. In this review, we summarise recent findings regarding the impact of nutrition on the variation in COVID-19 disease severity and also its potential impact on the control of the disease during the current pandemic. Understanding the dietary pattern that is deleterious to COVID-19 survival might help to improve public health strategies toward reducing the spread of COVID-19 and designing new approaches for control and maybe even treatment of this new disease.

## Pathogenesis of COVID-19 Disease

*S*ARS-CoV-2 virus primarily affects the respiratory system, although other organ systems are involved as well. Lower respiratory tract infection-related symptoms including fever, dry cough, and dyspnoea were reported in the initial case series from Wuhan, China (6). In addition, headache, dizziness, generalised weakness, vomiting and diarrhoea were observed (18). Although COVID-19 is mainly a respiratory disease, the gastrointestinal system can also act as a reservoir for SARS-CoV-2 (19). In addition; neurological manifestations are also reported in most hospitalised COVID-19 patients (20).

It is now widely recognised that the respiratory symptoms of COVID-19 are extremely heterogeneous, ranging from minimal symptoms to significant hypoxia with acute respiratory distress syndrome (ARDS) (8, 21). In the first reports from Wuhan, the time between the onset of symptoms and the development of ARDS was as short as 9 days, indicating that the respiratory symptoms could progress rapidly (6). ACE2is identified as a functional receptor for SARS-CoV-2 (22). Structural and functional analysis showed that the SARS-CoV-2 spike protein binds to the ACE2 receptor (23–25). ACE2 expression is high in the lung, heart, ileum, kidney and bladder (26). More specifically the ACE2 receptor is highly expressed on the apical side of lung epithelial cells in the alveolar space (27, 28). This correlates with the fact that early lung injury was often seen in the distal airways (29).

Genetic susceptibility can be a major factor in the host response to infectious diseases where inborn errors of the immune system are often critical (30). Differences in clinical outcomes of COVID-19 may also be determined by genetic susceptibility. Old age, gender and comorbidities including hypertension, diabetes, respiratory system disease and cardiovascular disease have all been identified as being closely associated with disease severity and mortality and represent significant risk factors (31).

COVID-19 morbidity and mortality rise dramatically with age and co-existing health conditions, including cancer and cardiovascular diseases. While most infected individuals recover, even very young, and otherwise healthy patients may unpredictably succumb to this disease (32). Questions still remain as to how susceptibility and outcome factors relate to SARS-CoV-2 infection.

In this line the greater severity of the disease was associated with maladapted immune responses and host ACE2. However, some other genetic parameters for SARS-CoV-2 receptor and entry gene expression and function have been described (33).

An intact immune system is essential for an effective defence against invading microorganisms. However, due to the immunological defects seen with COVD-19, there is reduced scope for a defence to be mounted against SARS-CoV-2 (34). The massive production of cytokines and chemokines observed during COVID-19 infection, the so-called “cytokine storm,” leads to broad and uncontrolled tissue damage and results in plasma leakage, enhanced vascular permeability and disseminated and vascular coagulation. This excessive proinflammatory host response is responsible for the pathological outcomes such as acute lung injury (ALI) and ARDS seen in severe SARS-CoV-2 patients, which typically leads to death.

Men are at a greater risk of severe symptoms and worse outcomes from COVID-19 than women. The precise reason for this discrepancy is not fully understood, but genetic factors, the effects of sex hormones such as oestrogen and testosterone as well as differences in immune cell function such as that of mast cells may be important factors (35).

Prostate cancer patients who were receiving androgen-deprivation therapy (ADT), a treatment that suppresses the production of androgens that fuels prostate cancer cell growth, had a significantly lower risk of SARS-CoV-2 infection (36). This suggests that blocking androgens in men is protective against SARS-CoV-2 infection. There is also evidence that males and females have different levels of receptors that recognise pathogens or that serve as an ingress point for SARS-CoV-2. Whilst there is currently no conclusive evidence for a role of ACE2 receptors and associated proteases being differentially expressed in males compared to females, it remains a potential contributing factor.

## Physical Inactivity, Malnutrition, and COVID-19

**Figure 1:**
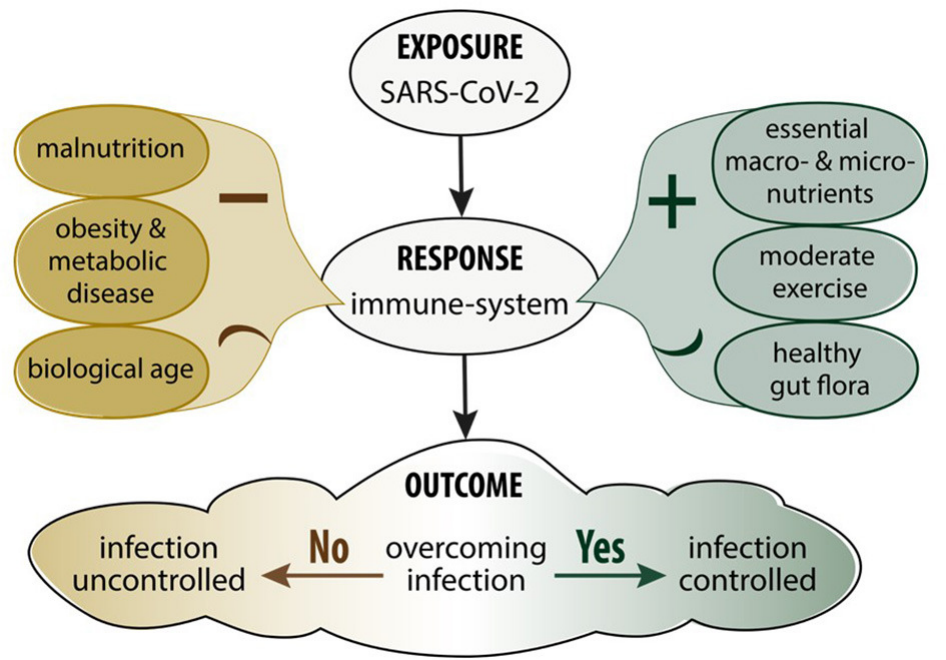
Impact of nutrition, metabolic disease and exercise on the immune response and SARS-CoV-2 infection.

Balanced nutrition is an important determinant in immune function against infectious disease in general (14). Poor nutrition and an unhealthy diet significantly weakens the immune system and increases susceptibility to infectious disease (37). A reduction in physical activity and a higher energy intake have been observed as a consequence of pandemic isolation measures which is especially worrisome since they both enhance the risk of a more severe outcome of COVID-19 (38). This is particularly true in middle-aged and elderly people where physical inactivity negatively impacts cardio-vascular functional capacity, body weight, metabolic function, muscle strength, haemostatic factors and immune functions (39). Moderate, but not vigorous exercise, enhances immune processes resulting in lower incidence of upper respiratory tract infections (39). Figure 1 summarises how levels of exercise and diet affect immune functions. A suboptimal diet may significantly affect the susceptibility to COVID-19 infection as well as the downstream consequences including severity, recovery and the potential for re-infection in different patient populations (40). Diets with a high consumption of saturated fatty acids (SFA), sugars, refined carbohydrates, and low levels of fibre and antioxidants modulate the balance between the adaptive and innate immune responses leading to an impaired host defence against viruses (41). In addition, these diets are associated with a higher prevalence of COVID-19 risk factors and the long term recovery from COVID-19 infection (42).

SFA-rich diets induce chronic activation of the innate immune system while inhibiting adaptive immunity. In fact, high SFA diets induce a lipotoxic state which could activate toll-like receptor (TLR) 4 on the surface of macrophages and neutrophils and lead to chronic activation of the innate immune system. This, in turn, may trigger other inflammatory signalling pathways and the production of proinflammatory mediators (41, 43). The expression levels of TLR9 and levels of endogenous triggers for TLR9 activation are also influenced by diet which has been proposed to contribute to a severe outcome of COVID-19 in vulnerable patients (44).

A high fat diet (HFD) and obesity increases TLR9 expression in visceral adipose tissue in mice and human (45). HFD induces excess production of nucleic acids and related protein antigens worsening metabolic inflammation through activation of macrophages and expansion of plasmacytoid dendritic cells (pDCs) in the liver (46). In animal models, HFD also increases macrophage infiltration into the lung tissue and alveoli. A similar process may underlie the high rate of inflammation in lung epithelial cells and the alveolar damage seen in obese COVID-19 patients or those with evidence of metabolic syndrome (47). Furthermore, carbohydrates, sugars and a HFD increase oxidative stress and thereby impair the proliferation and maturation of both B and T cells and induce apoptosis which together results in suppression of the adaptive immune response to viral infection (48).

In animal models of influenza infection, a HFD enhanced lung damage and delayed the onset of the adaptive immune response. This was associated with impaired memory T cell function and a reduced capacity to respond to antigen presentation and clearance of the influenza virus (48). The mechanism(s) causing the increased lung damage are unclear but may involve programmed cell death (49–52).

As a result, the elderly, patients with comorbidities, and those with risk factors for COVID-19 should be cautious with the consumption of unhealthy diets that could pose an increased risk to COVID-19 severity. A healthy, balanced diet should contain the necessary macro- and micronutrients, vitamins, minerals, and maybe even unique microbes such as probiotics that can restore and maintain immune function (53).

Proteins, vitamins and minerals have, for a long time, been considered important factors in health and resistance against infection due to their impact on immune homeostasis (54). The immune-effect of natural herbal medicines such as Shuang-Huang-Lian oral liquid during upper respiratory tract infections may be explained, at least in part, by specific proteins, and other active ingredients (2, 55). A recent comprehensive meta-analysis regarding the effect of nutrition status on the immune response to respiratory viral infection reported that vitamins and minerals play a determinant role in the ability to mount an immune defence against respiratory viral infection and are associated with the severity of infection outcome (56).

In the current COVID-19 pandemic, there are reports of vitamins and minerals affecting the severity of infection and mortality. For example, low prealbumin levels is associated with increased severity of ARDS in patients with SARS-CoV-2 (57). Vitamins A, B complex, C, D and E, and trace elements have an important role in the prolonged and effective stimulation of the immune system (58, 59). Thus, deficiencies in vitamin and trace element levels could result in a more detrimental fate in response to viral infections including SARS-Cov2 (60). Some studies also suggest beneficial effects of natural compounds.

In summary, the nutritional status of an individual has a significant impact on not only the susceptibility to, but also the severity of, COVID-19 infection. The next section provides additional details concerning the impact of proteins, vitamins and minerals in viral respiratory infections that might help finding new strategies for the prevention and control of SARS-CoV-2 infection (Table 1).

**Table 1:** Overall role and impact of nutrition on immune function.

		**Role and impact on immune responses**
Protein		1. Production of cytokines and antibodies. 2. Regulation of both humeral and cellular immunity specially Tell immunity. 3. Regulation of DNA replication and cell division. 4. Generation of nitric oxide, superoxide dismutase (SOD) and glutathione peroxidase (GSH-Px) as well as scavenging activity by immune cells.
Vitamins	A group vitamins	1. Antiviral immunity. 2. Regulation of the proliferation and differentiation of immune cells *via* nuclear retinoid acid receptor.
	B group vitamins	1. Immune metabolic pathways as co-factor. 2. Viral clearance *via* regulation of natural killer cells and cytotoxic CD8^+^ lymphocyte functions.
	C group vitamins	1. Act as enzymatic co-factor and an essential antioxidant in boosting immune functions including phagocytosis, cell signalling, antibody production leucocyte migration, and hormone production.
	D group vitamins	1. Controlling inflammation in the lungs. 2. Proliferation and activation of viral specific immune cells *via* its receptor. 3. Upregulation of cytokines and their recruitment to the infected sites.
	E group vitamins	1. Antioxidant activity. 2. Gene transcription of proteins involved in T-cell proliferation, phagocytosis and cytotoxicity, regulate the production of reactive oxygen species (ROS) and reactive nitrogen species (RNS), and modulate signal transduction.
Minerals	Zinc	1. Antiviral and antibacterial immunity, inhibition of viral RNA polymerase and ACE2 activity. 2. Involved in modulation of inflammatory cytokines. 3. Upregulation of Th1cytokine responses, activation of immune metabolic pathways.
	Selenium	1. Antioxidant and anti-inflammatory properties. 2. Increase in T-cell proliferation. 3. Upregulation of IL-10.
	Copper	1. Inhibition of viral replication and release. 2. Inhibition of viral-induced cell apoptosis. 3. Activity of ceruloplasmin, benzylamine oxidase and superoxide dismutase and improvement of the cell antioxidant status.
	Magnesium	1. Activator role in many of enzymatic reactions. 2. Regulation of nuclear factor*-κ*B, Il-6, c-reactive protein, and other related signalling pathways.
Probiotics		1. Influencing immune reactions by up or down regulation of immune responses

### Proteins

Proteins are critical factors in immune-nutrition and essential for the production of, for example, immunoglobulins, and cytokines. Dietary proteins are digested to their constituent amino acids and dietary protein deficiency reduces plasma concentrations of most amino acids. Amino acids, such as arginine are the precursor of polyamines that play a significant role in the regulation of DNA replication and cell division. In addition, optimal antibody production requires a sufficient plasma arginine level. Supplementation with arginine significantly increases T cell function as well as enhancing their numbers compared with control subjects (61). Furthermore, arginine is essential for the generation of nitric oxide by macrophages, an essential component of the innate immune response. In contrast, methionine has an important role in the growth, development and histological structure of immune organs and enhances macrophage phagocytic activity (62). Methionine deficiency also decreases lymphocyte activities and inhibits the proliferation and differentiation of B and T cells (63). Methionine also plays a role in both humeral and cellular immunity since methionine deficiency significantly affects antibody titre and decreases serum levels of IgG, IgA, and IgM. Furthermore, methionine deficiency decreases the relative percentage of CD3^+^, CD3^+^/CD8^+^, and CD3^+^/CD4^+^T lymphocytes (64). Given the importance of T cell immunity in the defence against COVID-19, this aspect of methionine deficiency is essential in the prevention of, and reduction in the severity of infection.

Reduction of sulphur-containing amino acids in the serum significantly reduces the hydroxyl radical scavenging activity of superoxide dismutase (SOD) and glutathione peroxidase (GSH-Px) which helps to protect the host against viral infection (3, 4). Thus, methionine deficiency can result in oxidative damage and lipid peroxidation, which will lead to a failure in cellular immunity.

Amino acids are also important components for cytokine production. The production of interleukin (IL)-1, IL-6 and tumour necrosis factor (TNF) α is strongly dependent on the metabolism of sulphur-containing amino acids including methionine and cysteine (65).

The effect of dietary proteins in improving immune function has been reported in cancer patients. In a clinical trial, whey protein isolate (WPI) enriched with Zn and Se improved cell-mediated immunity and antioxidant capacity in cancer patients undergoing chemotherapy. WPI is an alternative oral nutrition supplement (ONS) that contains high quality protein and amino acid profiles. WPI increases GSH function because of its cysteine-enriched supplementation, reduces oxidative free radical formation and prevents infection (5). This suggests that WPI supplementation may improve GSH levels and thereby enhance immunity in subjects at risk of COVID-19 as well as reducing the severity of the disease in patients already infected with SARS-CoV-2.

### Vitamins

A healthy immune system may aid the prevention and treatment of patients with COVID-19 (62). Vitamins play an important role in normal immune function and their dietary levels tightly regulate immune reactions (66) (Table 1). For example, vitamins A and D increased humeral immunity following influenza vaccination in children (63, 67). Fasted individuals are encouraged to have sufficient and timely intake of healthy and functional foods including vitamins in order to maintain exercise performance and immune function (17).

Vitamin A is an important player in the regulation of both the cellular and humoral arms of the immune system and significantly increased the antibody response after anti-viral vaccination (56). Vitamin A acts *via* the nuclear retinoid acid receptor (68, 69) and regulates the proliferation and differentiation of immune cells and modulates the expression of proinflammatory cytokines including TNFα and IL 6 (70, 71).

A protective role of vitamin A has been indicated against in a variety of lung infections, HIV, and malaria (72, 73). In animal models of corona virus infection, the levels of plasma retinol and retinol-binding protein is significantly reduced and mortality from respiratory infections decreases in those with adequate vitamin A within their diets (74, 75). As a result, we postulate that vitamin A supplementation may make a useful contribution in combating the risk of susceptibility to COVID-19 infection and reducing the severity of the disease in patients.

B group vitamins are key players in metabolic pathways particularly those of organic molecules. Furthermore, the important role of B group vitamins including folic acid, B12, and B6 in immune function is well known. For example, the active form of vitamin B6, pyridoxal phosphate, is a cofactor for many metabolic processes particularly transamination or breakdown of amino acids and the metabolism of important immunomodulatory mediators (76, 77). These metabolic pathways are also important in viral infection suggesting that a balance intake of these vitamins is necessary in the regulation of the viral immune response. In particular, they regulate the function of natural killer cells and cytotoxic CD8^+^ lymphocytes and thereby contribute to effective viral clearance (78).

Vitamin D is fat soluble and known as a multifunctional agent in a broad range of bodily functions including immune reactions (79). Vitamin D receptors (VDRs) are expressed in a broad range of respiratory epithelial and immune cells and vitamin D activation is induced by cytokines and TLRs within the respiratory tract (79, 80). Epidemiological studies indicated the importance of vitamin D in the immune defence against influenza A and B, parainfluenza and respiratory syncytial virus (RSV) (81, 82). Interestingly; low levels of serum vitamin D enhanced the risk of both upper and lower respiratory tract infections (83). It has been reported that serum vitamin D levels of ≥95 nmol/L significantly reduced the rate of acute viral respiratory tract infections two-fold (60).

On the other hand, low levels of vitamin D are associated with enhanced levels of inflammatory cytokines and an increase in the incidence of many diseases. Importantly, vitamin D deficiency is associated with increased thrombotic episodes, obesity, and diabetes which are frequently observed in severe COVID-19 patients (84). An inhibitory and antiviral activity of vitamin D in human nasal epithelial cells infected with SARS-CoV-2S has been reported (85).

Vitamin D deficiency has shown an important role in reducing the risk of severe disease and mortality in COVID-19 patients. In Chicago, more than half of COVID-19 related deaths occurred in African-American individuals known to have vitamin D deficiency (86). Indeed, regions with the highest rates of COVID-19 mortality are those with a high prevalence of vitamin D deficiency (66). Indeed, a meta-analysis indicates that low serum levels of vitamin D is significantly associated with the risk, seriousness and mortality of COVID-19 (87). Although the area is controversial, the limited current data suggests that higher serum vitamin D levels favour a decreased risk of COVID-19 infection and mortality (88). It is reasonable, therefore, to suggest that regular vitamin D supplementation would be of benefit to individuals at greater risk of infection or of developing severe disease (89).

Vitamin E is a potent regulator of host immune functions due to its antioxidant capacity. This enables vitamin E to modulate multiple immune and inflammatory responses including T-cell proliferation, granulocyte phagocytosis, and cytotoxicity through effects on gene transcription (90–93). This explains why vitamin E deficiency is accompanied by impairment of both humoral and cellular immunity (94). Although vitamin E supplementation increased the risk of pneumonia in smokers (95), vitamin E had a therapeutic benefit in chronic hepatitis B (HB) patients in a small pilot randomised clinical trial (RCT) (96). In another RCT, vitamin E treatment led to higher anti-HBe seroconversion in children (97). A computational analysis to assess the ability of FDA-approved drugs to block coronavirus binding to ACE2 or transmembrane protease, serine 2 (TMPRSS2) and downstream transcriptomic profiles indicated that vitamin E, ruxolitinib and glutamine were likely to significantly attenuate infection by SARS-CoV-2 (98). This needs to be confirmed in human studies.

Vitamin C boosts many aspects of the immune system including cell signalling, phagocytosis, antibody production, immune cells proliferation and leukocyte migration to the site of infection (99). Furthermore, vitamin C mediates many physiological events, such as hormone production and immune homeostasis and acts as an essential antioxidant and enzymatic co-factor in many cellular functions (58).

Animal studies highlight its role in improving the production of interferons (IFN)α and β in response to influenza A virus and this may explain its ability to protect against coronavirus infection (100). Indeed, higher serum levels of vitamin C is associated with a reduced incidence of pneumonia and lower respiratory tract infections (101, 102). In addition, vitamin C reduces the duration and severity of the common cold (58), and of upper respiratory tract infections (101).

Vitamin C also promotes the repair of the damaged tissues (58) and high-dose intravenous vitamin C has a beneficial effect in patients with virus-induced ARDS which results from severe lung damage (103). Since ARDS is evident in many subjects with severe COVID-19 it supports the concept that vitamin C may be useful in the treatment of COVID-19 (104). Further studies are required to demonstrate a link between COVID-19 incidence and severity with systemic vitamin C levels.

Interestingly, apart from individuals with impaired glucose 6-phosphate activity and renal failure, no adverse effects of large doses of intravenously or orally administrated vitamin C have been detected (105, 106).

### Minerals

In addition to vitamins, several minerals have a beneficial and supportive role in enhancing antiviral immune responses and thus could be beneficial in controlling COVID-19 (Table 1). Zinc plays a pivotal role in the immune system particularly in antiviral and antibacterial immunity (107). Zinc deficiency is associated with an increased susceptibility to infectious and viral diseases and studies have shown that the zinc status is a critical factor that can influence immunity against viral infections (108). In patients infected with torque tenovirus (TTV), injection of a high dose of zinc enhances the immune response (107). On the other hand, low-dose supplementation of zinc together with selenium improved the humoral immune response to influenza vaccine and increased antibody titres (109).

In *in vitro* experiments Zn inhibits the SARS-CoV-2 RNA polymerase (110). Interestingly, chloroquine that has some protective efficiency against coronaviruses acts as a zinc ionophore (111). In addition, zinc may supress ACE2 activity and regulate the production of IFNα to improve antiviral activity (108) and zinc also has an anti-inflammatory role by inhibiting NF-κB signalling (112) and modulating regulatory T-cell functions. This combination of actions may be important in sequencing the cytokine storm present in subjects with COVID-19 (112).

Zinc-deficient populations are at an increased risk of infection by several viruses including human immunodeficiency virus (HIV) and hepatitis C virus (HCV) (113). In a RCT, Zn increased Th1cytokine responses including the production of IL-2 and of INFγ in response to influenza vaccine (107). In another RCT, oral supplementation of high-dose zinc after stem cell transplantation demonstrated that zinc enhanced thymic function and the production of CD4 naïve T cells, helping to prevent the reactivation of TTV (107). However, in an elderly population, enhancing zinc plasma concentrations had no effect on the antibody response or on the number of lymphocytes present following influenza vaccination (114).

Selenium is another trace element with a broad range of effects from antioxidant to anti-inflammatory properties (115). Selenium supplementation resulted in both beneficial and detrimental effects on cellular immunity to influenza. Selenium supplemented subjects had a more rapid clearance of the poliovirus after vaccination for influenza. In this study, selenium induced a dose-dependent increase in T-cell proliferation and the production of IL-8 and IL-10. However, mucosal influenza-specific antibody responses were unaffected by selenium supplementation (116).

Copper has a crucial role in the development and differentiation of immune cells and mediates several antiviral responses (117). Chelates of thujaplicin and copper inhibited influenza virus-induced apoptosis *in vitro* supressed viral replication and release from the infected cells (118). In addition, intracellular copper interferes with the influenza virus life cycle (119). Appropriate copper intake optimises the antioxidant status and improves the serum level and activity of ceruloplasmin, benzylamine oxidase and superoxide dismutase (118, 120).

Magnesium also regulates immune function by controlling various aspects of immunity such as immunoglobulin synthesis and antibody-dependent cytolysis (121). Magnesium is an activator of many enzymatic reactions and is essential for a broad range of physiological functions (122). Magnesium intake supports different aspects of immune functions including regulation of NF-κB, IL-6, c-reactive protein, and other related signalling pathways (123). The major role of magnesium is in viral immunity which has been reported in many *in-vitro* and *in-vivo* studies (121, 124). A recent study reported that magnesium in combination with vitamin D and vitamin B_12._ Significantly reduce the proportion of severe COVID-19 patients needing intensive care (125).

### Probiotics

SARS-CoV-2 infection of the gastrointestinal system affects gut inflammation both directly and indirectly following infection of intestinal epithelial cells through the ACE2 and transmembrane protease serine 2 (TMPRSS2) viral entry system. This results in pronounced pro-inflammatory chemokine and cytokine release (126, 127). In addition, cellular and animal studies indicate that SARS-CoV-2 instigates an acute intestinal inflammatory response including elevated levels of faecal calprotectin and serum IL-6 and linked to clinical evidence of diarrhoea (127). To date, the rationale for using microbiome modulators such as pre and probiotics in COVID-19 is indirect. Two randomised controlled trials showed that critically ill patients on mechanical ventilation who were given probiotics (*Lactobacillus rhamnosus* GG, live *Bacillus subtilis*, and *Enterococcus faecalis*) developed substantially less ventilator-associated pneumonia compared with placebo (128, 129). Due to the similarities between severe COVID-19- and pneumonia-induced ARDS there is potential for this therapeutic approach being useful in COVID-19.

Patients with COVID-19 appear to have an altered gut microbiome with depletion of beneficial commensals (*Eubacterium ventriosum, Faecalibacterium prausnitzii, Roseburia, and Lachnospiraceae taxa*) and enrichment of opportunistic pathogens (*Clostridium hathewayi, Actinomyces viscosus, Bacteroides nordii*) (130). It is uncertain whether this difference is causal or downstream of other changes but again indicates that probiotics or microbiome manipulation may be useful in severe COVID-19 subjects. Disturbances in gut microbiota and their metabolites influence immune responses, inflammation and diseases of the lungs by mediating both over-active and under-active immune responses (131). Favourable implications of gut microbiota modulation in COVID-19 is speculated upon because a general imbalance of gut microbiota is commonly seen in elderly and immune-compromised patients and patients with other co-morbidities like type-2 diabetes, and cardiovascular disorders (132). To date however, the rationale for using probiotics in COVID-19 is derived from indirect evidence and more research is needed before any specific recommendations on probiotic use can be made (133).

## Conclusion

The COVID-19 pandemic poses a significant threat to humans. Until the widespread availability of effective, long-term, vaccines, and effective treatment and prevention measures. An important therapeutic and preventive strategy, may be to reduce the incidence or severity of infection. This will involve having a healthy and resilient immune system. An individual's nutritional status has a significant impact on the susceptibility to COVID-19, response to therapy, and on the long-lasting consequences of infection. As such, it is critical to consider the impact of lifestyle and the consumption of healthy diets during the pandemic.

A good healthy balanced nutrition is vital in the recovery process for all patients with COVID-19, particularly those who have suffered cardiac distress, pulmonary distress, or those who have been critically ill due to the weight loss, frailty or sarcopenia associated with these conditions (134). These patients require individually tailored nutrition support, started early in their journey, that is sufficient and timed to enable optimal metabolic utilisation to aid recovery (134). Nutritional rehabilitation needs to be central to the community management of these patients' post-hospital discharge to ensure efficient and effective recovery and to reduce the risk of hospital re-admissions or the duration of long-COVID-19.

In this respect, access to healthy foods should be a priority for individuals and governments to reduce the susceptibility and prolonged effects of COVID19. Given the over-representation of minorities with the disease and those who also have poor nutrition, we should aim to increase the access to healthy fresh food as well as provide nutritional education to these at-risk subjects.

## Author Contributions

EM and SA designed draft and wrote first version of manuscript draught. GB, GF, SM, JG, and IA revised and comments to the manuscript. All authors has seen and approved final version of manuscript.

## Conflict of Interest

The authors declare that the research was conducted in the absence of any commercial or financial relationships that could be construed as a potential conflict of interest.

## References

[1] Zhu N Zhang D Wang W Li X Yang B Song J. A novel coronavirus from patients with pneumonia in China, 2019. N Engl J Med. (2020) 382:727–33. 10.1056/NEJMoa2001017 31978945 PMC7092803

[2] Dousari AS Moghadam MT Satarzadeh N. COVID-19 (Coronavirus Disease 2019): a new coronavirus disease. Infect Drug Resist. (2020) 13:2819. 10.2147/IDR.S259279 32848431 PMC7429403

[3] Epidemiology Working Group for NCIP Epidemic Response Chinese Center for Disease Control and Prevention. The epidemiological characteristics of an outbreak of 2019 novel coronavirus diseases (COVID-19) in China. Zhonghua Liu Xing Bing Xue Za Zhi. (2020) 41:145. 10.46234/ccdcw2020.032 32064853

[4] Simon J Helter TM White RG van der Boor C Łaszewska A. Impacts of the Covid-19 lockdown and relevant vulnerabilities on capability well-being, mental health and social support: an Austrian survey study. BMC Public Health. (2021) 21:1–12. 10.1186/s12889-021-10351-5 33557816 PMC7868863

[5] Chen N Zhou M Dong X Qu J Gong F Han Y. Epidemiological and clinical characteristics of 99 cases of 2019 novel coronavirus pneumonia in Wuhan, China: a descriptive study. Lancet. (2020) 395:507–13. 10.1016/S0140-6736(20)30211-7 32007143 PMC7135076

[6] Huang C Wang Y Li X Ren L Zhao J Hu Y. Clinical features of patients infected with 2019 novel coronavirus in Wuhan, China. Lancet. (2020) 395:497–506. 10.1016/S0140-6736(20)30183-5 31986264 PMC7159299

[7] Xu X-W Wu X-X Jiang X-G Xu K-J Ying L-J Ma C-L. Clinical findings in a group of patients infected with the 2019 novel coronavirus (SARS-Cov-2) outside of Wuhan, China: retrospective case series. BMJ. (2020) 368:m606. 10.1136/bmj.m606 32075786 PMC7224340

[8] Mortaz E Tabarsi P Varahram M Folkerts G Adcock IM. The immune response and immunopathology of COVID-19. Front Immunol. (2020) 11:2037. 10.3389/fimmu.2020.02037 32983152 PMC7479965

[9] Moghadam MT Babakhani S Rajabi S Baravati FB Raeisi M Dousari AS. Does stress and anxiety contribute to COVID-19? Iran J Psychiatry Behav Sci.. (2020) 15:e106041. 10.5812/ijpbs.106041

[10] Alipoor SD Mortaz E Jamaati H Tabarsi P Bayram H Varahram M. COVID-19: molecular and cellular response. Front. Cell. Infect. Microbiol. (2021) 11:563085. 10.3389/fcimb.2021.563085 33643932 PMC7904902

[11] Mohammad S Aziz R Al Mahri S Malik SS Haji E Khan AH. Obesity and COVID-19: what makes obese host so vulnerable? Immun Ageing. (2021) 18:1–10. 10.1186/s12979-020-00212-x 33390183 PMC7779330

[12] Pérez LM Pareja-Galeano H Sanchis-Gomar F Emanuele E Lucia A Gálvez BG. ‘Adipaging': ageing and obesity share biological hallmarks related to a dysfunctional adipose tissue. J Physiol. (2016) 594:3187–207. 10.1113/JP271691 26926488 PMC4908019

[13] Rao S Lau A So H-C. Exploring diseases/traits and blood proteins causally related to expression of ACE2, the putative receptor of SARS-CoV-2: a Mendelian randomization analysis highlights tentative relevance of diabetes-related traits. Diabetes Care. (2020) 43:1416–26. 10.2337/dc20-0643 32430459

[14] Curtis LJ Bernier P Jeejeebhoy K Allard J Duerksen D Gramlich L. Costs of hospital malnutrition. Clin Nutr. (2017) 36:1391–6. 10.1016/j.clnu.2016.09.009 27765524

[15] Wadhera RK Wadhera P Gaba P Figueroa JF Maddox KEJ Yeh RW. Variation in COVID-19 hospitalizations and deaths across New York City boroughs. JAMA. (2020). 323:2192–5. 10.1001/jama.2020.7197 32347898 PMC7191469

[16] Bousquet J Anto JM Iaccarino G Czarlewski W Haahtela T Anto A. Is diet partly responsible for differences in COVID-19 death rates between and within countries? ClinTrans Allergy. (2020) 10:16. 10.1186/s13601-020-00351-w 32499909 PMC7250534

[17] Moghadam MT Taati B Paydar Ardakani SM Suzuki K Ramadan fasting during the COVID-19 pandemic; observance of health nutrition and exercise criteria for improving the immune system. Front Nutr. (2021) 7:349. 10.3389/fnut.2020.570235 PMC7838371 33521030

[18] Shi H Han X Jiang N Cao Y Alwalid O Gu J. Radiological findings from 81 patients with COVID-19 pneumonia in Wuhan, China: a descriptive study. Lancet Infect Dis. (2020) 20:425–34. 10.1016/S1473-3099(20)30086-4 32105637 PMC7159053

[19] Ng SC Tilg H. COVID-19 and the gastrointestinal tract: more than meets the eye. Gut. (2020) 69:973–4. 10.1136/gutjnl-2020-321195 32273292 PMC7211058

[20] Mao L Jin H Wang M Hu Y Chen S He Q. Neurologic manifestations of hospitalized patients with coronavirus disease 2019 in wuhan, china. JAMA Neurol. (2020) 77:683–90. 10.1001/jamaneurol.2020.1127 32275288 PMC7149362

[21] Alipoor SD Jamaati H Tabarsi P Mortaz E. Immunopathogenesis of Pneumonia in COVID-19. Tanaffos. (2020) 19:79. 33262791 PMC7680509

[22] Li W Moore MJ Vasilieva N Sui J Wong SK Berne MA. Angiotensin-converting enzyme 2 is a functional receptor for the SARS coronavirus. Nature. (2003) 426:450–4. 10.1038/nature02145 14647384 PMC7095016

[23] Chen Y Guo Y Pan Y Zhao ZJ. Structure analysis of the receptor binding of 2019-nCoV. Biochem Biophys Res Commun. (2020) 525:135–40. 10.1016/j.bbrc.2020.02.071 32081428 PMC7092824

[24] Walls AC Park Y-J Tortorici MA Wall A McGuire AT Veesler D. Structure, function, and antigenicity of the SARS-CoV-2 spike glycoprotein. Cell. (2020) 181:281–92.e6. 10.1016/j.cell.2020.02.058 32155444 PMC7102599

[25] Letko M Marzi A Munster V. Functional assessment of cell entry and receptor usage for SARS-CoV-2 and other lineage B betacoronaviruses. Nature Microbiol. (2020) 5:562–9. 10.1038/s41564-020-0688-y 32094589 PMC7095430

[26] Zou X Chen K Zou J Han P Hao J Han Z. Single-cell RNA-seq data analysis on the receptor ACE2 expression reveals the potential risk of different human organs vulnerable to 2019-nCoV infection. Front Med. (2020) 14:185–92. 10.1007/s11684-020-0754-0 32170560 PMC7088738

[27] Hamming I Timens W Bulthuis M Lely A Navis Gv van Goor H. Tissue distribution of ACE2 protein, the functional receptor for SARS coronavirus. A first step in understanding SARS pathogenesis. J Pathol. (2004) 203:631–7. 10.1002/path.1570 15141377 PMC7167720

[28] Jia HP Look DC Shi L Hickey M Pewe L Netland J. ACE2 receptor expression and severe acute respiratory syndrome coronavirus infection depend on differentiation of human airway epithelia. J Virolo. (2005) 79:14614–21. 10.1128/JVI.79.23.14614-14621.2005 16282461 PMC1287568

[29] Yoshikawa T Hill T Li K Peters CJ Tseng C-TK. Severe acute respiratory syndrome (SARS) coronavirus-induced lung epithelial cytokines exacerbate SARS pathogenesis by modulating intrinsic functions of monocyte-derived macrophages and dendritic cells. J Virol. (2009) 83:3039–48. 10.1128/JVI.01792-08 19004938 PMC2655569

[30] Dutta S Thakare YR Kshirsagar A Sarkar D. A review on host genetic susceptibility to SARS CoV-2 related pneumonia. Int J Pharm Sci. (2021) 12:b42–49. 10.22376/ijpbs.2021.12.2.b42-49

[31] Gemmati D Bramanti B Serino ML Secchiero P Zauli G Tisato V. COVID-19 and individual genetic susceptibility/receptivity: role of ACE1/ACE2 genes, immunity, inflammation and coagulation. might the double X-chromosome in females be protective against SARS-CoV-2 compared to the single X-chromosome in males? Int J Mol Sci. (2020) 21:3474. 10.3390/ijms21103474 32423094 PMC7278991

[32] Dong Y Mo X Hu Y Qi X Jiang F Jiang Z. Epidemiology of COVID-19 among children in China. Pediatrics. (2020) 145:e20200702. 10.1542/peds.2020-0702 32179660

[33] Wu A Peng Y Huang B Ding X Wang X Niu P. Genome composition and divergence of the novel coronavirus (2019-nCoV) originating in China. Cell Host Microbe. (2020) 27:325–8. 10.1016/j.chom.2020.02.001 32035028 PMC7154514

[34] Babaha F Rezaei N. Primary immunodeficiency diseases in COVID-19 pandemic: a predisposing or protective factor? AJMS. (2020) 360:740–1. 10.1016/j.amjms.2020.07.027 32773108 PMC7388814

[35] Conti P Younes A. Coronavirus COV-19/SARS-CoV-2 affects women less than men: clinical response to viral infection. J Biol Regul Homeost Agents. (2020) 34:71. 10.23812/Editorial-Conti-3 32253888

[36] Montopoli M Zumerle S Vettor R Rugge M Zorzi M Catapano CV. Androgen-deprivation therapies for prostate cancer and risk of infection by SARS-CoV-2: a population-based study (n= 4532). Ann Oncol. (2020) 31:1040–5. 10.1016/j.annonc.2020.04.479 32387456 PMC7202813

[37] Childs CE Calder PC Miles EA. Diet and immune function. Nutrients. (2019) 11:1933. 10.3390/nu11081933 PMC6723551 31426423

[38] Gallo LA Gallo TF Young SL Moritz KM Akison LK. The impact of isolation measures due to COVID-19 on energy intake and physical activity levels in Australian university students. Nutrients. (2020) 12:1865. 10.3390/nu12061865 32585830 PMC7353248

[39] Bull FC Hardman AE. Walking: a best buy for public and planetary health. Br J Sports Med. (2018) 52:755–756. 10.1136/bjsports-2017-098566 29187348

[40] Jayawardena R Misra A. Balanced diet is a major casualty in COVID-19. Diabetes Metab Syndr. (2020) 14:1085–6. 10.1016/j.dsx.2020.07.001 32652495 PMC7333608

[41] Milanski M Degasperi G Coope A Morari J Denis R Cintra DE. Saturated fatty acids produce an inflammatory response predominantly through the activation of TLR4 signaling in hypothalamus: implications for the pathogenesis of obesity. J Neurosci. (2009) 29:359–70. 10.1523/JNEUROSCI2760-08.2009 19144836 PMC6664935

[42] Butler MJ Barrientos RM. The impact of nutrition on COVID-19 susceptibility and long-term consequences. Brain Behav Immun. (2020) 87:53–4. 10.1016/j.bbi.2020.04.040 32311498 PMC7165103

[43] Rogero MM Calder PC. Obesity, inflammation, toll-like receptor 4 and fatty acids. Nutrients. (2018) 10:432. 10.3390/nu10040432 29601492 PMC5946217

[44] Bezemer GF Garssen J. TLR9 and COVID-19: a multidisciplinary theory of a multifaceted therapeutic target. Front Pharmacol. (2020) 11:601685. 10.3389/fphar.2020.601685 33519463 PMC7844586

[45] Thomalla M Schmid A Neumann E Pfefferle PI Müller-Ladner U Schäffler A. Evidence of an anti-inflammatory toll-like receptor 9 (TLR 9) pathway in adipocytes. J Endocrinol. (2019) 240:325–43. 10.1530/JOE-18-0326 30508414

[46] Revelo XS Ghazarian M Chng MHY Luck H Kim JH Zeng K. Nucleic acid-targeting pathways promote inflammation in obesity-related insulin resistance. Cell Rep. (2016) 16:717–30. 10.1016/j.celrep.2016.06.024 27373163 PMC6354586

[47] Costa Dias M Joyce R Postel-Vinay F Xu X. The challenges for labour market policy during the Covid-19 pandemic. Fis Stud. (2020) 41:371–82. 10.1111/1475-5890.12233 32836541 PMC7361552

[48] Green WD Beck MA. Obesity impairs the adaptive immune response to influenza virus. Ann Am Thorac Soc. (2017) 14(Suppl. 5):S406–9. 10.1513/AnnalsATS.201706-447AW 29161078 PMC5711276

[49] Shubina M Tummers B Boyd DF Zhang T Yin C Gautam A. Necroptosis restricts influenza A virus as a stand-alone cell death mechanism. J Exp Med. (2020) 217:e20191259. 10.1084/jem.20191259 32797196 PMC7596817

[50] Zheng M Kanneganti TD. The regulation of the ZBP1-NLRP3 inflammasome and its implications in pyroptosis, apoptosis, and necroptosis (PANoptosis). Immunol Rev. (2020) 297:26–38. 10.1111/imr.12909 32729116 PMC7811275

[51] Wang Y Hao Q Florence JM Jung B-G Kurdowska AK Samten B. Influenza virus infection induces ZBP1 expression and necroptosis in mouse lungs. Front Cell Infect Microbiol. (2019) 9:286. 10.3389/fcimb.2019.00286 31440477 PMC6694206

[52] Zhang T Yin C Boyd DF Quarato G Ingram JP Shubina M. Influenza virus Z-RNAs induce ZBP1-mediated necroptosis. Cell. (2020) 180:1115–29.e13. 10.1016/j.cell.2020.02.050 32200799 PMC7153753

[53] Zabetakis I Lordan R Norton C Tsoupras A. COVID-19: the inflammation link and the role of nutrition in potential mitigation. Nutrients. (2020) 12:1466. 10.3390/nu12051466 32438620 PMC7284818

[54] Wintergerst ES Maggini S Hornig DH. Contribution of selected vitamins and trace elements to immune function. Ann NutrMetab. (2007) 51:301–23. 10.1159/000107673 17726308

[55] Shi H Han X Zheng C. Evolution of CT manifestations in a patient recovered from 2019 novel coronavirus (2019-nCoV) pneumonia in Wuhan, China. Radiol. (2020) 295:20. 10.1148/radiol.2020200269 32032497 PMC7233359

[56] Jayawardena R Sooriyaarachchi P Chourdakis M Jeewandara C Ranasinghe P. Enhancing immunity in viral infections, with special emphasis on COVID-19: a review. Diabetes Metab Syndr. (2020) 14:367–82. 10.1016/j.dsx.2020.04.015 32334392 PMC7161532

[57] Zuo P Tong S Yan Q Cheng L Li Y Song K. Decreased prealbumin level is associated with increased risk for mortality in elderly hospitalized patients with COVID-19. Nutrition. (2020) 78: 110930. 10.1016/j.nut.2020.110930 32854020 PMC7333599

[58] Carr AC Maggini S. Vitamin C and immune function. Nutrients. (2017) 9: 1211. 10.3390/nu9111211 PMC5707683 29099763

[59] Martineau AR Jolliffe DA Hooper RL Greenberg L Aloia JF Bergman P. Vitamin D supplementation to prevent acute respiratory tract infections: systematic review and meta-analysis of individual participant data. BMJ. (2017) 356:i6583. 10.1136/bmj.i6583 28202713 PMC5310969

[60] Merzon E Tworowski D Gorohovski A Vinker S Golan Cohen A Green I. Low plasma 25 (OH) vitamin D level is associated with increased risk of COVID-19 infection: an Israeli population-based study. FEBS J. (2020) 287:3693–702. 10.1111/febs.15495 32700398 PMC7404739

[61] Kim S-H Roszik J Grimm EA Ekmekcioglu S. Impact of l-arginine metabolism on immune response and anticancer immunotherapy. Front Oncol. (2018) 8:67. 10.3389/fonc.2018.00067 29616189 PMC5864849

[62] Jia T1 FH Sun J Zhang Y Yang W Li Y. Foxp3 expression in A549 cells is regulated by Toll-like receptor 4 through nuclear factor-κB. Mol Med Rep. (2012) 6:167–72. 10.3892/mmr.2012.877 22576743

[63] Chockalingam AK Hamed S Goodwin DG Rosenzweig BA Pang E Boyne MT II. The effect of Oseltamivir on the disease progression of lethal influenza A virus infection: plasma cytokine and miRNA responses in a mouse model. Dis Markers. (2016) 2016: 9296457. 10.1155/2016/9296457 27110056 PMC4824134

[64] Zhu M Ruan T Zeng Q Wu B. Effects of methionine deficiency on the B lymphocyte and immunoglobulins of Cecal tonsil in Cobb broilers. Braz J Poult Sci. (2019) 21:1–8. 10.1590/1806-9061-2019-1059

[65] Tesseraud S Coustard SM Collin A Seiliez I. Role of sulfur amino acids in controlling nutrient metabolism and cell functions: implications for nutrition. Br JNutr. (2008) 101:1132–9. 10.1017/S0007114508159025 19079841

[66] Martineau AR Forouhi NG. Vitamin D for COVID-19: a case to answer? Lancet Diabetes Endocrinol. (2020) 8:735–6. 10.1016/S2213-8587(20)30268-0 32758429 PMC7398646

[67] Patel N Penkert RR Jones BG Sealy RE Surman SL Sun Y. Baseline serum vitamin A and D levels determine benefit of oral vitamin A&D supplements to humoral immune responses following pediatric influenza vaccination. Viruses. (2019) 11:907. 10.3390/v11100907 31575021 PMC6832482

[68] Evans RM Mangelsdorf DJ. Nuclear receptors, RXR, and the big bang. Cell. (2014) 157:255–66. 10.1016/j.cell.2014.03.012 24679540 PMC4029515

[69] Di Masi A Leboffe L De Marinis E Pagano F Cicconi L Rochette-Egly C. Retinoic acid receptors: from molecular mechanisms to cancer therapy. Mol Aspects Med. (2015) 41:1–115. 10.1016/j.mam.2014.12.003 25543955

[70] Villamor E Mbise R Spiegelman D Hertzmark E Fataki M Peterson KE. Vitamin A supplements ameliorate the adverse effect of HIV-1, malaria, and diarrheal infections on child growth. Pediatrics. (2002) 109:e6. 10.1542/peds.109.1.e6 11773574

[71] Aukrust P Müller F Ueland T Svardal A Berge R Frøland S. Decreased vitamin A levels in common variable immunodeficiency: vitamin A supplementation in vivo enhances immunoglobulin production and downregulates inflammatory responses. Eur J Clin Invest. (2000) 30:252–9. 10.1046/j.1365-2362.2000.00619.x 10692003

[72] Glasziou P Mackerras D. Vitamin A supplementation in infectious diseases: a meta-analysis. BMJ. (1993) 306: 366–70. 10.1136/bmj.306.6874.366 8461682 PMC1676417

[73] Wu T Ni J Wei J. Vitamin A for non-measles pneumonia in children. Cochrane Database Syst Rev. (2005) 2005:CD00370. 10.1002/14651858.CD003700.pub2 PMC6991929 16034908

[74] West CE Sijtsma SR Kouwenhoven B Rombout JH van der Zijpp AJ. Epithelia-damaging virus infections affect vitamin A status in chickens. J Nutr. (1992) 122:333–9. 10.1093/jn/122.2.333 1310111

[75] Semba RD. Vitamin A and immunity to viral, bacterial and protozoan infections. PNAS. (1999) 58:719–27. 10.1017/S0029665199000944 10604208

[76] Percudani R Peracchi A. The B6 database: a tool for the description and classification of vitamin B6-dependent enzymatic activities and of the corresponding protein families. BMC Bioinformat. (2009) 10:1–8. 10.1186/1471-2105-10-273 19723314 PMC2748086

[77] Bourquin F Capitani G Grütter MG. PLP-dependent enzymes as entry and exit gates of sphingolipid metabolism. Protein Sci. (2011) 20:1492–508. 10.1002/pro.679 21710479 PMC3190145

[78] Mikkelsen K Stojanovska L Prakash M Apostolopoulos V. The effects of vitamin B on the immune/cytokine network and their involvement in depression. Maturitas. (2017) 96: 58–71. 10.1016/j.maturitas.2016.11.012 28041597

[79] Teymoori-Rad M Shokri F Salimi V Marashi SM. The interplay between vitamin D and viral infections. Rev Med Virol. (2019) 29: e2032. 10.1002/rmv.2032 30614127

[80] Mok CK Ng YL Ahidjo BA Lee RCH Loe MWC Liu J. Calcitriol, the active form of vitamin D, is a promising candidate for COVID-19 prophylaxis. bioRxiv. (2020). 10.1101/2020.06.21.162396

[81] Gruber-Bzura BM. Vitamin D and influenza—prevention or therapy? Int J Mol Sci. (2018) 19: 2419. 10.3390/ijms19082419 PMC6121423 30115864

[82] Jiménez-Sousa MÁ Martínez I Medrano LM Fernández-Rodríguez A Resino S. Vitamin D in human immunodeficiency virus infection: influence on immunity and disease. Front Immunol. (2018) 9: 458. 10.3389/fimmu.2018.00458 29593721 PMC5857570

[83] Hurwitz JL Jones BG Penkert RR Gansebom S Sun Y Tang L. Low retinol-binding protein and vitamin D levels are associated with severe outcomes in children hospitalized with lower respiratory tract infection and respiratory syncytial virus or human metapneumovirus detection. J Pediatr. (2017) 187:323–7. 10.1016/j.jpeds.2017.04.061 28578159 PMC5588918

[84] Grant WB Lahore H McDonnell SL Baggerly CA French CB Aliano JL. Evidence that vitamin D supplementation could reduce risk of influenza and COVID-19 infections and deaths. Nutrients. (2020) 12: 988. 10.3390/nu12040988 32252338 PMC7231123

[85] Jolliffe DA Griffiths CJ Martineau AR. Vitamin D in the prevention of acute respiratory infection: systematic review of clinical studies. J Steroid BiochemMol Biol. (2013) 136:321–9. 10.1016/j.jsbmb.2012.11.017 23220552

[86] Meltzer DO Best TJ Zhang H Vokes T Arora V Solway J. Association of vitamin D status and other clinical characteristics with COVID-19 test results. JAMA Network Open. (2020) 3: e2019722. 10.1001/jamanetworkopen.2020.19722 32880651 PMC7489852

[87] Yisak H Ewunetei A Kefale B Mamuye M Teshome F Ambaw B. Effects of vitamin D on COVID-19 infection and prognosis: a systematic review. Risk Manag Healthc Policy. (2021) 14: 31. 10.2147/RMHPS291584 33447107 PMC7800698

[88] Teshome A Adane A Girma B Mekonnen ZA. The impact of vitamin D level on COVID-19 infection: systematic review and meta-analysis. Front Public Health. (2021) 9:169. 10.3389/fpubh.2021.624559 33748066 PMC7973108

[89] Boulkrane MS Ilina V Melchakov R Fedotova J Drago F Gozzo L. COVID-19 disease and vitamin D: a mini-review. Front Pharmacol. (2020). 11: 2107. 10.3389/fphar.2020.604579 33390994 PMC7773655

[90] Wu D Meydani SN. Vitamin E, immune function, and protection against infection. In: Vitamin E in Human Health. Springer. (2019) p. 371–84. 10.1007/978-3-030-05315-4_26

[91] Lewis ED Meydani SN Wu D. Regulatory role of vitamin E in the immune system and inflammation. IUBMB Life. (2019) 71:487–94. 10.1002/iub.1976 30501009 PMC7011499

[92] Marko MG Ahmed T Bunnell SC Wu D Chung H Huber BT. Age-associated decline in effective immune synapse formation of CD4+ T cells is reversed by vitamin E supplementation. J Immunol. (2007) 178:1443–9. 10.4049/jimmunol.178.3.1443 17237392

[93] Han SN Pang E Zingg J-M Meydani SN Meydani M Azzi A. Differential effects of natural and synthetic vitamin E on gene transcription in murine T lymphocytes. Arch Biochem Biophy. (2010) 495:49–55. 10.1016/j.abb.2009.12.015 20026030

[94] Moriguchi S Muraga M. Vitamin E and immunity. (2000) 59:305–36. 10.1016/S0083-6729(00)59011-6 10714244

[95] Hemilä H Kaprio J. Vitamin E supplementation and pneumonia risk in males who initiated smoking at an early age: effect modification by body weight and dietary vitamin C. Nutrition. (2008). 7:33. 10.1186/1475-2891-7-33 19019244 PMC2603040

[96] Andreone P Fiorino S Cursaro C Gramenzi A Margotti M Di Giammarino L. Vitamin E as treatment for chronic hepatitis B: results of a randomized controlled pilot trial. Antiviral Res. (2001) 49:75–81. 10.1016/S0166-3542(00)00141-8 11248360

[97] Fiorino S Bacchi-Reggiani ML Leandri P Loggi E Andreone P. Vitamin E for the treatment of children with hepatitis B e antigen-positive chronic hepatitis: a systematic review and meta-analysis. World J Hepatol. (2017) 9:333. 10.4254/wjh.v9.i6.333 28293383 PMC5332423

[98] Kim J Zhang J Cha Y Kolitz S Funt J Chong RE. Advanced bioinformatics rapidly identifies existing therapeutics for patients with coronavirus disease-2019 (COVID-19). J Trans Med. (2020) 18:1–9. 10.1186/s12967-020-02430-9 32586380 PMC7315012

[99] Field CJ IR Johnson PD Schley. Nutrients and their role in host resistance to infection. J Leukoc Biol. (2002) 71:16–32. 11781377

[100] Atherton J Kratzing C Fisher A. The effect of ascorbic acid on infection of chick-embryo ciliated tracheal organ cultures by coronavirus. Arch Virol. (1978) 56:195–9. 10.1007/BF01317848 205194 PMC7087159

[101] Hemilä H. Vitamin C intake and susceptibility to pneumonia. Pediatr Infect Dis J. (1997) 16:836–7. 10.1097/00006454-199709000-00003 9306475

[102] Hemilä H. Vitamin C and SARS coronavirus. J Antimicrob Chemother. (2003) 52:1049–50. 10.1093/jac/dkh002 14613951 PMC7110025

[103] Fowler AA III Kim C Lepler L Malhotra R Debesa O Natarajan R. Intravenous vitamin C as adjunctive therapy for enterovirus/rhinovirus induced acute respiratory distress syndrome. World J Crit Care Med. (2017) 6:85. 10.5492/wjccm.v6.i1.85 28224112 PMC5295174

[104] Carr AC. A new clinical trial to test high-dose vitamin C in patients with COVID-19. Crit Care. (2020) 24:1–2. 10.1186/s13054-020-02851-4 32264963 PMC7137406

[105] Cathcart RF. Vitamin C, titrating to bowel tolerance, anascorbemia, and acute induced scurvy. Medical Hypotheses. (1981) 7:1359–76. 10.1016/0306-9877(81)90126-2 7321921

[106] Padayatty SJ Sun AY Chen Q Espey MG Drisko M Levine. Vitamin C: intravenous use by complementary and alternative medicine practitioners and adverse effects. PLoS ONE. (2010) 5:e11414. 10.1371/journal.pone.0011414 20628650 PMC2898816

[107] Iovino L Mazziotta F Carulli G Guerrini F Morganti R Mazzotti V. High-dose zinc oral supplementation after stem cell transplantation causes an increase of TRECs and CD4+ naive lymphocytes and prevents TTV reactivation. Leuk Res. (2018) 70:20–4. 10.1016/j.leukres.2018.04.016 29747074

[108] Skalny AV Rink L Ajsuvakova OP Aschner M Gritsenko VA Alekseenko SI. Zinc and respiratory tract infections: Perspectives for COVID-19. Int J Mol Med. (2020) 46:17–26. 10.3892/ijmm.2020.4575 32319538 PMC7255455

[109] Girodon F Galan P Monget A-L Boutron-Ruault M-C Brunet-Lecomte P Preziosi P. Impact of trace elements and vitamin supplementation on immunity and infections in institutionalized elderly patients: a randomized controlled trial. Arch Intern Med. (1999) 159:748–54. 10.1001/archinte.159.7.748 10218756

[110] Sodhi M Etminan M. Therapeutic potential for tetracyclines in the treatment of COVID-19. Pharmacotherapy. (2020) 40:487–8. 10.1002/phar.2395 32267566 PMC7262278

[111] Derwand R Scholz M. Does zinc supplementation enhance the clinical efficacy of chloroquine/hydroxychloroquine to win todays battle against COVID-19? Med Hypotheses. (2020) 142:109815. 10.1016/j.mehy.2020.109815 32408070 PMC7202847

[112] Jarosz M Olbert M Wyszogrodzka G Młyniec K Librowski T. Antioxidant and anti-inflammatory effects of zinc. Zinc-dependent NF-κB signaling. Inflammopharmacol. (2017) 25:11–24. 10.1007/s10787-017-0309-4 28083748 PMC5306179

[113] Read SA Obeid S Ahlenstiel C Ahlenstiel G. The role of zinc in antiviral immunity. Adv Nutr. (2019) 10:696–710. 10.1093/advances/nmz013 31305906 PMC6628855

[114] Provinciali M Montenovo A STEFANO GD Colombo M Daghetta L Cairati M. Effect of zinc or zinc plus arginine supplementation on antibody titre and lymphocyte subsets after influenza vaccination in elderly subjects: a randomized controlled trial. Age Ageing. (1998) 27:715–22. 10.1093/ageing/27.6.715 10408666

[115] Rayman MP. Selenium and human health. Lancet. (2012) 379:1256–68. 10.1016/S0140-6736(11)61452-9 22381456

[116] Ivory K Prieto E Spinks C Armah CN Goldson AJ Dainty JR. Selenium supplementation has beneficial and detrimental effects on immunity to influenza vaccine in older adults. Clinical Nutrition. (2017) 36:407–15. 10.1016/j.clnu.2015.12.003 26803169 PMC5381341

[117] Li C Li Y Ding C. The role of copper homeostasis at the host-pathogen axis: from bacteria to fungi. Int J Mol Sci. (2019) 20:175. 10.3390/ijms20010175 30621285 PMC6337107

[118] Miyamoto D Kusagaya Y Endo N Sometani A Takeo S Suzuki T. Thujaplicin–copper chelates inhibit replication of human influenza viruses. Antivir Res. (1998) 39:89–100. 10.1016/S0166-3542(98)00034-5 9806486

[119] Rupp JC Locatelli M Grieser A Ramos A Campbell PJ Yi H. Host cell copper transporters CTR1 and ATP7A are important for influenza A virus replication. Virol J. (2017) 14:1–12. 10.1186/s12985-016-0671-7 28115001 PMC5259989

[120] Turnlund JR Jacob RA Keen CL Strain J Kelley DS Domek JM. Long-term high copper intake: effects on indexes of copper status, antioxidant status, and immune function in young men. Am J Clin Nutr. (2004) 79:1037–44. 10.1093/ajcn/79.6.1037 15159234

[121] Liang R-y Wu W Huang J Jiang S-p Lin Y. Magnesium affects the cytokine secretion of CD4+ T lymphocytes in acute asthma. J Asthma. (2012) 49:1012–5. 10.3109/02770903.2012.739240 23134345

[122] Tang C-F Ding H Jiao R-Q Wu X-X Kong L-D. Possibility of magnesium supplementation for supportive treatment in patients with COVID-19. Eur J Pharmacol. (2020) 886:173546. 10.1016/j.ejphar.2020.173546 32931782 PMC7486870

[123] Wallace TC. Combating COVID-19 and building immune resilience: a potential role for magnesium nutrition? J Am Coll Nutr. (2020) 39:685–93. 10.1080/07315724.2020.1785971 32649272

[124] Chaigne-Delalande B Li F-Y O'Connor GM Lukacs MJ Jiang P Zheng L. Mg2+ regulates cytotoxic functions of NK and CD8 T cells in chronic EBV infection through NKG2D Science. (2013) 341:186–91. 10.1126/science.1240094 23846901 PMC3894782

[125] Tan CW Ho LP Kalimuddin S Cherng BPZ Teh YE Thien SY. Cohort study to evaluate effect of vitamin D, magnesium, and vitamin b12 in combination on severe outcome progression in older patients with coronavirus (COVID-19). Nutrition. (2020) 79:111017. 10.1016/j.nut.2020.111017 33039952 PMC7832811

[126] Effenberger M Grabherr F Mayr L Schwaerzler J Nairz M Seifert M. Faecal calprotectin indicates intestinal inflammation in COVID-19. Gut. (2020) 69:1543–4. 10.1136/gutjnl-2020-321388 32312790 PMC7211078

[127] Zhang H Z Kang H Gong D Xu J Wang Z Li. Digestive system is a potential route of COVID-19: an analysis of single-cell coexpression pattern of key proteins in viral entry process. Gut. (2020) 69:1010–8. 10.1136/gutjnl-2020-320953

[128] Morrow LE Kollef MH Casale TB. Probiotic prophylaxis of ventilator-associated pneumonia: a blinded, randomized, controlled trial. Am J Respir Crit Care Med. (2010) 182:1058–64. 10.1164/rccm.200912-1853OC 20522788 PMC2970846

[129] Zeng J Wang C-T Zhang F-S Qi F Wang S-F Ma S. Effect of probiotics on the incidence of ventilator-associated pneumonia in critically ill patients: a randomized controlled multicenter trial. Intensive Care Med. (2016) 42:1018–28. 10.1007/s00134-016-4303-x 27043237

[130] Zuo T Zhang F Lui GC Yeoh YK Li AY Zhan H. Alterations in Gut microbiota of patients with COVID-19 during time of hospitalization. Gastroenterol. (2020) 159:944–55.e8. 10.1053/j.gastro.2020.05.048 32442562 PMC7237927

[131] Donati Zeppa S Agostini D Gervasi M Annibalini G Amatori S Ferrini F. Mutual interactions among exercise, sport supplements and microbiota. Nutrients. (2020) 12:17. 10.3390/nu12010017 31861755 PMC7019274

[132] Dhar D Mohanty A. Gut microbiota and Covid-19-possible link and implications. Virus Res. (2020) 2020:198018. 10.1016/j.virusres.2020.198018 32430279 PMC7217790

[133] Mak JW Chan FK Ng SC. Probiotics and COVID-19: one size does not fit all. Lancet Gastroenterol Hepatol. (2020) 5:644–45. 10.1016/S2468-1253(20)30122-9 32339473 PMC7182525

[134] Herridge MS Cheung AM Tansey CM Matte-Martyn A Diaz-Granados N Al-Saidi F. One-year outcomes in survivors of the acute respiratory distress syndrome. N Eng J Med. (2003) 348:683–93. 10.1056/NEJMoa022450 12594312

